# Genome-Wide Identification of the *TCP* Gene Family and Functional Analysis of *Gypsophila paniculata GpTCP10* in Regulating Organ Development of Transgenic *Arabidopsis*

**DOI:** 10.3390/plants15060949

**Published:** 2026-03-19

**Authors:** Yue Xu, Guoping Zhang, Huameng Huang, Mingdong Ran, Hongjia Zhang, Kang Luo, Chao Song, Xiaowei Yu, Lijuan Ding, Leifeng Zhao, Yun Zheng

**Affiliations:** 1 Yunnan International Joint Laboratory of Durian Functional Genomics, College of Landscape and Horticulture, Yunnan Agricultural University, Kunming 650500, Chinaranmingdong2025@163.com (M.R.); zhjzhanghongjia@163.com (H.Z.); lk1689252548@163.com (K.L.); songchao43992025@163.com (C.S.);; 2 College of Tobacco Science, Yunnan Agricultural University, Kunming 650201, China; huanghm7429@163.com; 3 College of Big Data, Yunnan Agricultural University, Kunming 650201, China; 19112759370@163.com

**Keywords:** *Gypsophila paniculata*, *TCP* family, *GpTCP10*, organ development, flower size, transgenetic *Arabidopsis thaliana*

## Abstract

TCP transcription factors constitute a key regulatory family in plants, playing crucial roles in plant growth and development. Although this gene family has been extensively studied across diverse plant species, research in *Gypsophila paniculata* remains limited. Through genome-wide identification and analysis, this study identified 17 *GpTCP* in *G. paniculata*. Our analysis revealed that all GpTCP proteins contain a conserved TCP domain, with subcellular localization predictions indicating nuclear localization. Promoter analysis identified multiple *cis*-regulatory elements associated with plant organ development and growth regulation. Chromosomal synteny studies showed that gene expansion within the *G. paniculata TCP* gene family occurred after subfamily differentiation. Over-expression of *GpTCP10* in *Arabidopsis thaliana* caused root development inhibition, leaf curling, smaller flowers, and yellowing of flowers. Further studies showed that in two normally growing *G. paniculata* varieties with different flower sizes, *GpTCP10* was specifically expressed in leaf and floral tissues, with significantly higher expression levels in the smaller-flowered *G. paniculata*. These findings reveal the evolutionary characteristics of the *TCP* family in *G. paniculata*, and highlight the role of *GpTCP10* in regulating organ growth and development in transgenic *Arabidopsis thaliana* and floral organ size in *G. paniculata*.

## 1. Introduction

The TEOSINTE BRANCHED 1/CYCLOIDEA/PROLIFERATING CELL FACTOR (TCP) gene family is a plant-specific transcription factor family that has undergone significant functional diversification while remaining highly conserved throughout plant evolution. Its defining characteristic is the conserved TCP domain, which regulates gene expression through DNA binding and protein–protein interactions, thereby contributing to multiple levels of plant development, from organelle differentiation to organogenesis [1]. The *TCP* gene family is named after its three founding members: *TB1*, a branching regulator in maize (*Zea mays*); *CYC*, a floral symmetry regulator in snapdragon (*Antirrhinum majus*); and *PCF*, cell proliferation-related genes in rice (*Oryza sativa*) [2,3]. Since their discovery, *TCP* genes have attracted considerable research interest due to their pivotal roles in plant morphogenesis, particularly their significant contributions to crop domestication (such as the improvement of plant architecture in maize) and the regulation of organ morphology [4,5].

The *TCP* gene family is widely distributed across land plants, from bryophytes to angiosperms [6,7]. The strong conservation of its core domain suggests that its ancestral functions were established early in plant evolution, whereas structural diversification has driven the emergence of functional diversity [1,8,9]. The *Arabidopsis thaliana* genome encodes 24 *TCP* members [3], *Oryza sativa* encodes 22 [10], and *Opisthopappus taihangensis* encodes 14 [11], with interspecific variation in gene number reflecting expansion and contraction events during family evolution. In recent years, advances in structural biology and genomics have progressively clarified the molecular regulatory networks governed by *TCP* genes [5]. Their multidimensional regulatory paradigm—spanning “from organelles to organs”—provides a new perspective for understanding the integrative nature of plant development [5].

TCP transcription factors play a central regulatory role in flower morphology and floral organ development in model plants and various horticultural crops. It is reported that *TCP* transcription factor family plays a central role in the proper growth and development of floral organs by participating in the regulation of multiple processes, including the cell cycle [2,12]. For example, *Erycina pusilla*, ectopic expression of the *CIN-like* gene *EpTCP11* alters petal growth, transforming radial symmetry into bilateral symmetry [13]. In the ornamental plant *Torenia fournieri*, *TfTCP8* and *TfTCP13* were shown to influence petal size by regulating cell proliferation [14]. More extensive studies indicate that *TCP* genes precisely control floral development by regulating downstream target gene networks, such as those involving floral organ characteristics, cell cycle, and hormone signaling [15,16,17]. However, despite *Gypsophila paniculata* being an important cut-flower crop where flower form and size are key ornamental traits, functional studies of its *TCP* genes remain absent, and the molecular regulatory mechanisms remain unclear.

The baby’s breath (*Gypsophila paniculata*) is one of the world’s most important cut flowers, belonging to the same family as carnations. The size of ornamental flowers is directly related to their ornamental characteristics and economic value [18,19]. In addition, the plants of *G. paniculata* contain various bioactive compounds with potential medicinal values, such as flavonoids, triterpene saponins, sterols, and volatiles, which increase the economic value [20,21]. During flower development, the flower size is mainly influenced by the number or size of the petal epidermal cells [22,23,24]. This involves cell division and cell expansion in the floral meristem [12,25]. At the same time, the size of the floral organs themselves is also regulated by genes that define organ boundaries during the early stages of flower bud formation.

Among the current cultivated varieties, the genetic determinants of flower size in *G. paniculata*, are of significant interest due to their impact on ornamental value and commercial cultivation [26]. Furthermore, morphological observations reveal that flower size varies among cultivars, with “Huixing 1” exhibiting larger flowers compared to “Cloudstar 4” [27]. Such differences imply underlying genetic factors that control floral dimensions. The flowers of *G. paniculata* are generally small, and are typically white with five lobes, but variations in size among cultivars point to genetic diversity influencing these traits [28]. In previous studies, research on *G. paniculata* has mainly focused on tissue culture and metabolite composition, with limited investigation into molecular mechanisms [29,30]. In recent research, the whole genome of wild-type *G. paniculata* was reported, revealing that the formation of double petals in *G. paniculata* is related to miR172-AP2, while its insensitivity to ethylene metabolism may be related to an early doubling event in evolution [31]. Since the double-flowered cultivars of *G. paniculata* are highly male-sterile, it is impossible to breed new varieties through traditional hybridization methods [32]. Therefore, investigating the molecular mechanisms underlying flower size in *G. paniculata* can provide guidance for molecular breeding.

In order to reveal the potential roles of *GpTCP10* in the flower development of *G. paniculata*, we systematically identified *TCP* genes in *G. paniculata*, performated phylogenetic analysis for the *TCP* genes in *G. paniculata* and several other species, and analyzed the *cis*-regulatory elements in the promoters of these *TCP* genes. Furthermore, we performed functional analysis of *GpTCP10* by tranfecting it to *Arabidopsis thaliana*. Compared to the wild-type, the over-expression of *GpTCP10* caused abnormal developments of several organs, such as flowers, leaves and roots. In two cultivars of *G. paniculata* with significantly different sizes of flowers, *GpTCP10* also showed significantly different expression levels. These results demonstrated that *GpTCP10* was an important gene in regulating flower development of *G. paniculata*.

## 2. Results

### 2.1. Identification and Physicochemical Propreties of GpTCP Genes

We identified TCP proteins from the *G. paniculata* protein database and analyzed the domains of the TCP proteins (see Materials and Methods for details). In total, 17 *TCP* family genes from *G. paniculata* were identified (Table 1), i.e., *Gpan01g00759*, *Gpan01g00196*, *Gpan01g00724*, *Gpan03g00539*, *Gpan03g01596*, *Gpan06g00621*, *Gpan08g01206*, *Gpan08g01775*, *Gpan08g01151*, *Gpan09g00267*, *Gpan11g00693*, *Gpan11g00727*, *Gpan12g00867*, *Gpan15g00412*, *Gpan16g00434*, *Gpan16g00380* and *Gpan17g00108*.

The physicochemical properties of the identified TCP proteins were listed in Table 1. Amino acid lengths of these *GpTCPs* ranged from 185 to 466 aa, the relative molecular weight varied between 19.21 and 49.01 kDa, and the theoretical pI (pH value at zero charge) values ranged from 4.90 to 9.40. The instability index ranged from 37.30 to 69.92, and the hydrophobicity index ranged from −1.208 to −0.401, and the aliphatic indices varied between 49.44 and 71.32. None of the GpTCP proteins possessed signal peptides or transmembrane domains, and subcellular localization predictions indicated that all GpTCP proteins were localized to the nucleus.

### 2.2. Multiple Sequence Alignment and Phylogenetic Tree Analysis of GpTCP Proteins

**Figure 1:**
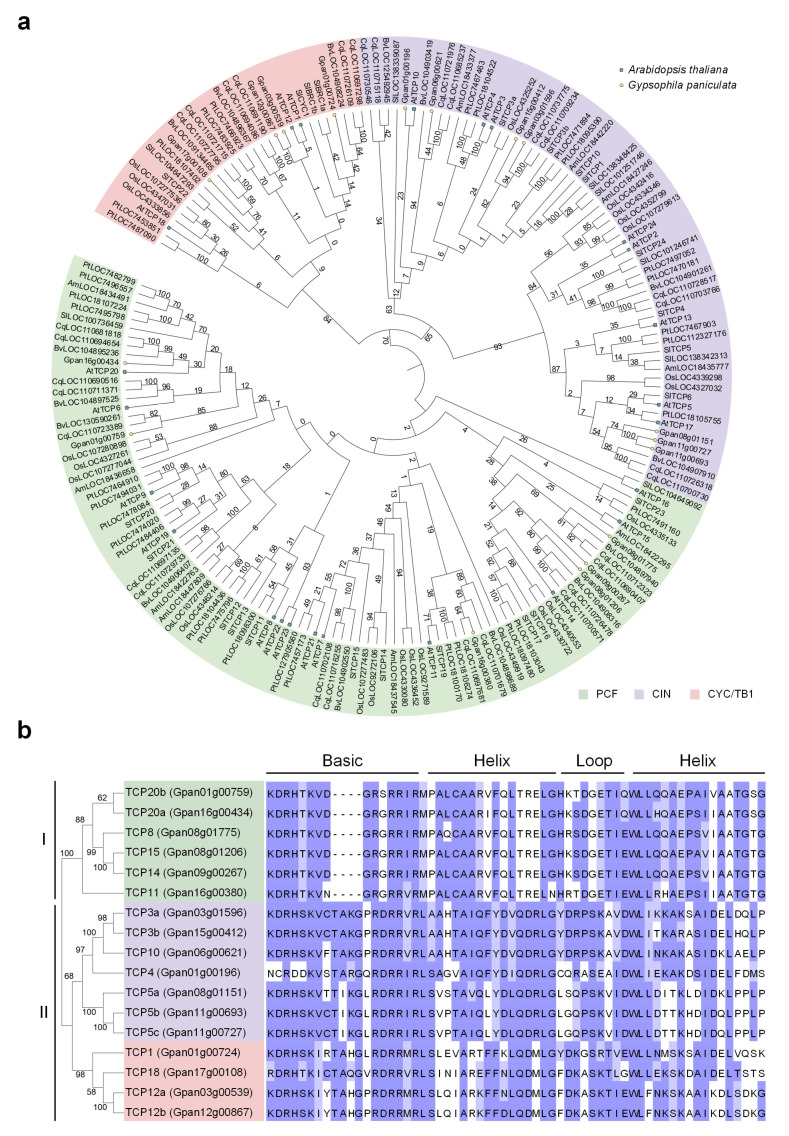
Conserved domain and evolutionary analysis of GpTCP proteins. (**a**) Phylogenetic analysis of TCP proteins from *Gypsophila paniculata*, *Arabidopsis thaliana*, *Amborella trichopoda*, *Beta vulgaris*, *Chenopodium quinoa*, *Oryza sativa*, *Populus trichocarpa*, *Solanum lycopersicum*. The phylogenetic tree was constructed using the Maximum Likelihood method, with 1000 bootstrap replicates, using MEGA (v12). (**b**) Multiple sequence alignment of GpTCP proteins. The phylogenetic tree was constructed using the Neighbor-Joining method, with 1000 bootstrap replicates, using MEGA (v12). In Part (**b**), the darker the purple, the more conserved the amino acid is.

The 17 TCP proteins identified from *G. paniculata*, along with a total of 185 TCP proteins from *A. thaliana*, *Amborella trichopoda*, *Beta vulgaris*, *Chenopodium quinoa*, *Oryza sativa*, *Populus trichocarpa*, and *Solanum lycopersicum*, were constructed into a phylogenetic tree. Our results revealed that *G. paniculata* TCP proteins shared closer evolutionary relationships with those from *B. vulgaris* and *C. quinoa*, all belonging to the Caryophyllales order. Within Class II, *G. paniculata* TCP proteins also exhibited relatively closer evolutionary relationships with *S. lycopersicum* TCP proteins, confirming the closer evolutionary ties between *G. paniculata* and core eudicots (Figure 1a).

The phylogenetic tree constructed using the Neighbor-Joining (NJ) method clustered 17 GpTCP proteins into two major branches: 6 proteins belonging to Class I (PCF) and 11 proteins belonging to Class II (Figure 1b). Combined with subfamily classification features, members were further divided into three subfamilies: PCF, CIN, and CYC/TB1 (labeled with green, purple, and pink blocks, respectively) (Figure 1a,b).

Additionally, multiple sequence alignment of the Basic-Helix-Loop-Helix region in TCP proteins revealed that Class I members exhibited extremely high sequence conservation in the Basic domain, Helix domain, and Loop region, such as the conserved Basic domain motif “KDRHTKVD” (Figure 1b). Although Class II members exhibited domain sequence variations between subtypes, they retained core conserved elements of the bHLH structure, for example, hydrophobic amino acid residues in the Helix region (Figure 1b). This feature aligned with the functional characteristics of the TCP family, which mediated DNA binding and protein interactions through the bHLH domain.

### 2.3. Conserved Motif Analysis and Promoter Cis-Element Analysis of GpTCPs

**Figure 2:**
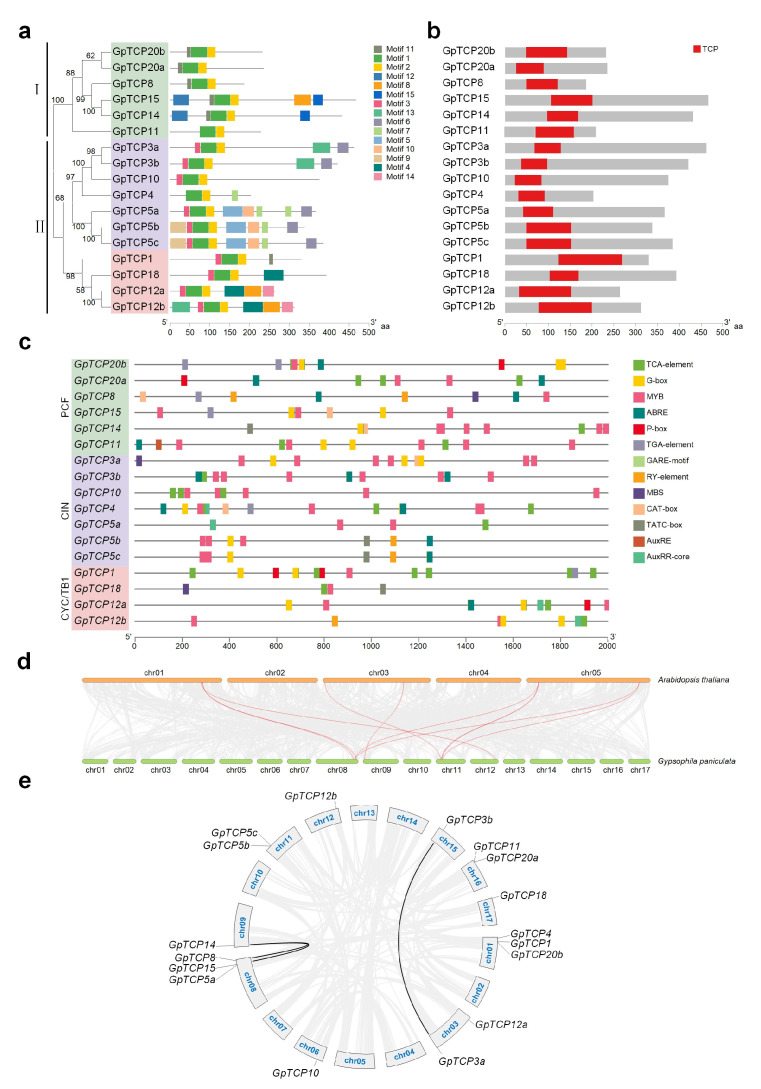
Analysis of conserved motifs in GpTCP proteins, *cis*-regulatory elements in their promoters, and chromosomal loci of the *GpTCP* genes. (**a**,**b**) Conserved motifs and domains of GpTCPs, respectively. Motifs 1–15 are represented by differently colored boxes, and the box length represents the motif length. The conserved TCP domain is indicated by a red box. (**c**) Analysis of *cis*-regulatory elements in the *CpTCP* promoters. (**d**) Synteny analysis of *Gypsophila paniculata* with *Arabidopsis thaliana*. (**e**) Chromosomal locations and synteny of *GpTCP* genes.

Conservative motif analysis revealed that GpTCP proteins possessed 15 distinct motifs, with each protein containing 2–7 conserved motifs and all harboring Motif 1 (Figure 2a). Results indicated that in Class I proteins, the core motifs were Motif 1/2/11 (GpTCP11 contained only Motif 1/2) and in Class II proteins, the core motifs were Motif 1/2/3. Motif 3 was unique to Class II proteins and served as a distinguishing feature between the two classes (Figure 2a). Within Class II proteins, Motifs 5/6/7/9/10/13 were specific to CIN proteins, while Motifs 4/8/14 were specific to CYC/TB1 proteins (Figure 2a). Domain analysis revealed that each GpTCP protein contained a TCP domain and were concentrated in the N-terminal region of the protein (Figure 2b). In Class I proteins, the lengths of structural domains were relatively consistent, whereas in Class II proteins, there were some variations in domain lengths (Figure 2b).

Analysis of *cis*-regulatory elements in the promoter region revealed 11 distinct *cis*-regulatory elements within the GpTCP promoter region, primarily involved in plant organ development and related hormone responses (Figure 2c). Among promoters of the 17 *GpTCP* gene, MYB transcription factor-associated recognition and binding sites (MYB and MBS) were present and constituted the most abundant elements (Figure 2c). Numerous *cis*-regulatory elements associated with plant hormone responses were also identified, including auxin-response elements (TGA-element, AuxRE, and AuxRR-core), abscisic acid (ABA)-response elements (ABREs), and gibberellin-response elements (GARE-motif, TATC-box and P-box) and salicylic acid (SA)-response elements (TCA-element) (Figure 2c). The promoter regions of multiple *GpTCP* genes contained G-box *cis*-regulatory elements, which were involved in various plant hormone signals, light signals, and stress signals. The promoter of *GpTCP5b/5c/8/12b* contained the RY-element *cis*-regulatory element, which was a seed-specific regulatory component. In addition, the promoter of *GpTCP4/8/11/14/15* contained the CAT-box *cis*-regulatory element, which was associated with meristem expression during plant growth and development (Figure 2c).

### 2.4. Chromosomal Colinearity Analysis of TCP Genes in A. thaliana and G. paniculata

Chromosomal colinearity analysis of *TCP* genes in *A. thaliana* and *G. paniculata* revealed that the two species share 10 homologous gene pairs, that demonstrated the functional core of the *TCP* family is conserved in angiosperms. (Figure 2d). Chromosomal colinearity analysis of *TCP* genes in *G. paniculata* revealed strong correlations between *GpTCP3a* on chromosome 03 and *GpTCP3b* on chromosome 15, both belonging to the *CIN* subfamily. Simultaneously, *GpTCP14* on chromosome 09 showed strong correlations with both *GpTCP8* and *GpTCP15* on chromosome 08, with all three genes belonging to the PCF subfamily (Figure 2d). This indicated that the two gene groups, *GpTCP3a/3b* and *GpTCP8/14/15*, might originate from the same ancestral gene. Through genomic duplication events, such as whole-genome duplication or segmental duplication, the gene families expanded. The presence of duplicated gene pairs within both the *PCF* and *CIN* subfamilies suggested that duplication events likely occurred after subfamily divergence. Each subfamily independently underwent gene duplication, doubling the numbers of gene members within them. In contrast, no strongly correlated genes were identified within the *CYC/TB1* subfamily, indicating that the evolutionary trajectories of genes within this subfamily were relatively independent (Figure 2d).

### 2.5. Regulation of Early Root Development by GpTCP10 in Arabidopsis

**Figure 3:**
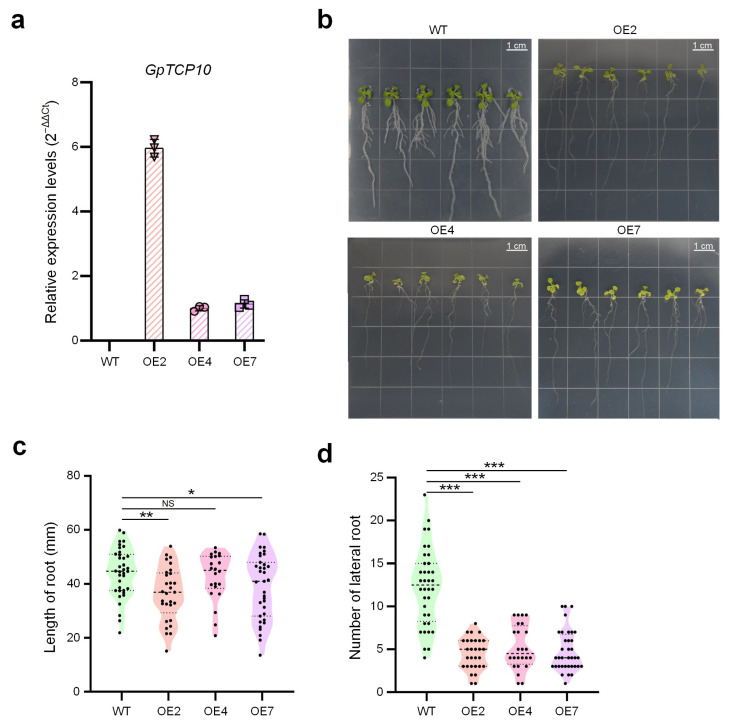
Regulation of early root development by *GpTCP10* in transgenic *Arabidopsis*. (**a**) Expression of *GpTCP10* gene in different *Arabidopsis* lines. WT: wild type; OE2/4/7: *GpTCP10* transgenic *Arabidopsis* line. (**b**) The roots of WT and *GpTCP10* over-expressing *Arabidopsis thaliana* lines. (**c**) Comparisons of root lengths at the day 13 from seedings of *GpTCP10* over-expressing transgenic and WT *Arabidopsis* lines. The numbers of samples in different groups are as following, WT: *n* = 36, OE2: *n* = 29, OE4: *n* = 22, and OE7: *n* = 35. (**d**) Comparisons of the numbers of lateral roots at the day 13 from seedings of *GpTCP10* over-expressing transgenic and WT *Arabidopsis* lines. The numbers of samples in different groups are as following, WT: *n* = 36, OE2: *n* = 29, OE4: *n* = 24, and OE7: *n* = 36. In Part (**c**,**d**), the dotted lines represent quantile lines; and the dashed lines represent median lines. In Part (**c**,**d**), “NS”, “*”, “**”, and “***” represent not significant, p< 0.05, 0.01, and 0.001, respectively, *t*-tests for Part (**a**,**c**) and Wilcoxon rank-sum test for Part (**d**). In Part (**a**), values are presented as the means ± standard deviations. The source data of Part (**a**,**c**,**d**) are available in Supplementary Tables S3, S4 and S5, respectively.

To investigate the functional role of *GpTCP10*, *GpTCP10* over-expressing transgenic *Arabidopsis* plants were generated by transforming the pCAMBIA1391 vector containing the 35S promoter. Validation of the T2-generation transgenic *Arabidopsis* lines was performed via qRT-PCR, with the three over-expression lines OE2, OE4, and OE7 selected for subsequent phenotypic observation. Among these, the OE2 line exhibited the highest expression level of *GpTCP10* (Figure 3a). Phenotypic observations of the T3 generation revealed that at 13 days post-emergence, the roots of the *GpTCP10* over-expressing lines were significantly thinner and weaker compared to those of the wild-type (Figure 3b). Except for the OE4 line, the root lengths of *GpTCP10* over-expressing lines at this stage were significantly shorter than those of the wild-type (Figure 3c). Additionally, statistical analysis of lateral root numbers indicated that *GpTCP10* over-expressing lines exhibited a significantly reduced number of lateral roots compared to the wild-type (Figure 3d).

### 2.6. Regulation of Early Leaf Development by GpTCP10 in Arabidopsis

**Figure 4:**
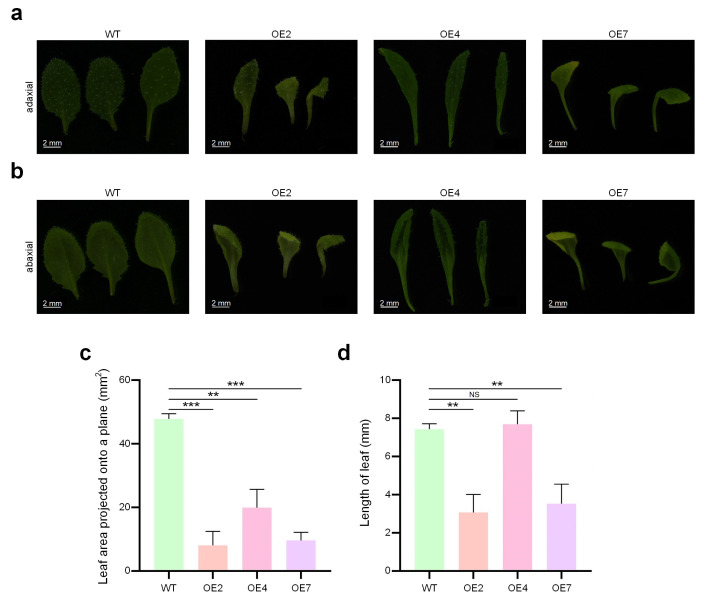
Regulation of leaf development by *GpTCP10* in transgenic *Arabidopsis*. (**a**) The adaxial views of leaves of WT and *GpTCP10* over-expression *Arabidopsis* lines. (**b**) The abaxial views of leaves of WT and *GpTCP10* over-expression *Arabidopsis* lines. (**c**) Comparisons of projected areas of the adaxial leaves of WT and *GpTCP10* over-expression *Arabidopsis* lines. (**d**) Comparisons of lengths of the primary leaf veins on abaxial leaves at the day 30 from seedings of *GpTCP10* over-expressing transgenic and WT *Arabidopsis* lines. In Part (**c**,**d**), “NS”, “**”, and “***” represent not significant, p< 0.01, and 0.001, respectively, *t*-tests. The data of each group are presented as the mean values ± standard deviations. The source data of Part (**c**,**d**) are available in Supplementary Tables S6 and S7, respectively.

Compared to wild-type *Arabidopsis*, the *GpTCP10* over-expression lines also exhibited a more curled leaf phenotype (Figure 4a,b). We also calculated the orthographic projection areas of leaves. Our results showed that the over-expressing lines had significantly smaller orthographic projection areas of leaves than the wild type (Figure 4c). Except for OE4, lengths of the primary leaf veins were also significantly shorter than those of the wild type (Figure 4d).

### 2.7. Regulation of Flower Size by GpTCP10 in Arabidopsis and G. paniculata

**Figure 5:**
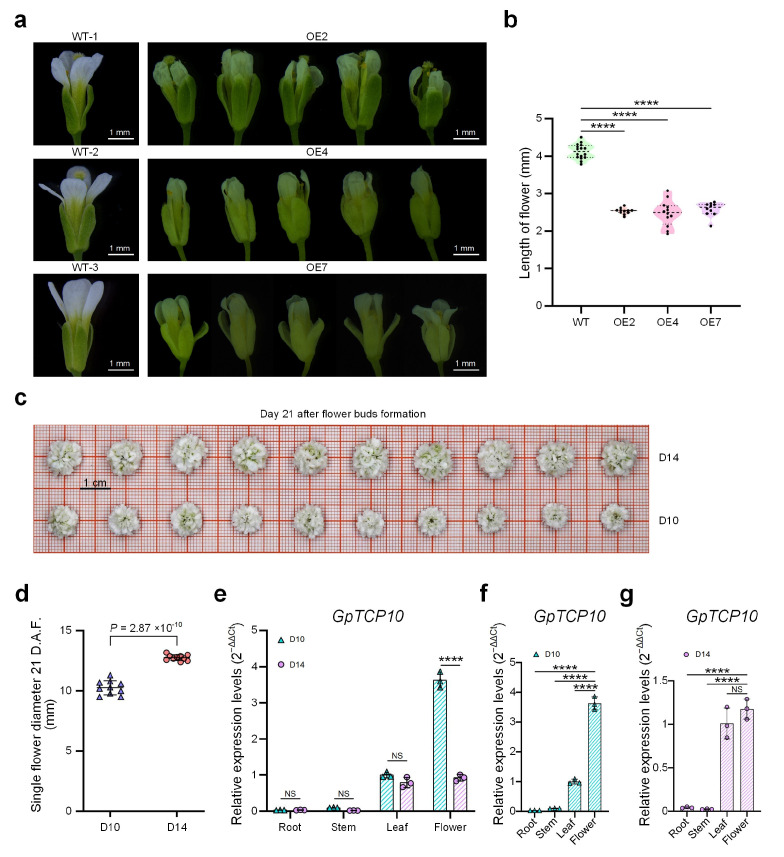
Regulation of flower size by *GpTCP10* in *Arabidopsis* and *Gypsophila paniculata*. (**a**) The flowers of WT and *GpTCP10* over-expressing transgenic *Arabidopsis thaliana*. (**b**) Comparisons of flower lengths at the day 30 from seedings of *GpTCP10* over-expressing transgenic and WT *Arabidopsis* lines. The numbers of samples in different groups are as following, WT: *n* = 16, OE2: *n* = 11, OE4: *n* = 12, and OE7: *n* = 10. (**c**) Flowers of two cultivated varieties of *Gypsophila paniculata* at day 21 after flower bud formation. D10: *Gypsophila paniculata* with individual flowers averaging about 10 mm in diameter. D14: *Gypsophila paniculata* with individual flowers averaging about 14 mm in diameter. (**d**) Comparisons of single flower diameters at day 21 after flower bud formation for D10 and D14. D.A.F.: day after flower bud formation. The numbers of samples in different *Gypsophila paniculata*, D10: *n* = 10, and D14: *n* = 10. (**e**) Relative expression of *GpTCP10* in different tissues of two *Gypsophila paniculata* cultivars, i.e., D10 and D14. (**f**) Relative expression of *GpTCP10* in different tissues of D10. (**g**) Relative expression of *GpTCP10* in different tissues of D14. In Part (**b**), the dotted lines represent quantile lines; and the dashed lines represent median lines. In part (**d**–**g**), expression levels were analyzed by qRT-PCR, with *GpActin* used as the internal reference gene. Data are presented as mean ± standard error (SE). In Part (**b**,**d**–**g**), “NS”, and “****” represent not significant and p< 0.0001, respectively, *t*-tests. In Part (**b**,**d**–**g**), the data of each group are presented as the mean ± standard deviation. The source data of Part (**b**,**d**–**g**) are available in Supplementary Tables S8, S9–S12, respectively.

The *GpTCP10* over-expressing lines exhibited a smaller flower size phenotype, with petals showing distinct wrinkling (Figure 5a). Overall, the color of floral organs appeared more yellowish compared to the wild type (Figure 5a). Measurements of petal length revealed significantly shorter petals in the *GpTCP10*-overexpression line compared to the wild type (Figure 5b).

To further investigate the effect of *GpTCP10* on petal size phenotype in *G. paniculata*, we identified two cultivated *G. paniculata* varieties exhibiting marked differences in flower sizes. As shown in Figure 5c,d, the diameters of D14 was significantly larger than those of D10. Only in floral tissues, the relative expression levels of the *GpTCP10* gene showed significant differences between D10 and D14 (Figure 5e). Moreover, *GpTCP10* expression was significantly higher in floral tissues than in other tissues of the D10 cultivar (Figure 5f), consistent with the smaller floral organ phenotype observed in *Arabidopsis* upon *GpTCP10* over-expression (Figure 5a–e). Among D14 cultivars, *GpTCP10* exhibited the highest expression levels across four tissues, though the differences compared to leaf expression were not significant (Figure 5g). The qRT-PCR results indicated that during normal growth and development in both cultivars, *GpTCP10* expression remained relatively low in roots, stems, and leaves in D10 and D14, respectively, (Figure 5f and Figure 5g, respectively). When *GpTCP10* was over-expressed throughout the whole *Arabidopsis* plant, we observed shorter, thinner roots (Figure 3b) and abnormally curled leaves (Figure 4a,b). This further indicated that *GpTCP10*, as a transcription factor, might not be primarily involved in root and leaf development under normal growth conditions in *G. paniculata*. Similarly, *GpTCP10* was specifically expressed in floral tissues and showed significantly higher expression in D10 flowers than in D14 flowers (Figure 5e), suggesting that *GpTCP10* was an important regulatory factor limiting floral organ expansion.

## 3. Discussion

The TCP plant-specific transcription factor plays a crucial role in organ growth and development in plants. We identified 17 *GpTCP* genes from the *G. paniculata* genome, with 6 belonging to the Class I *PCF* subgroup and II to Class II—7 of which belong to the *CIN* clade and 4 to the *CYC/TB1* clade (Figure 1b). The number of *TCP* gene family members varied across species [7]. Compared to *Arabidopsis* [33], which possessed 13 *PCF*, 8 *CIN*, and 3 *CYC/TB1* subgroup members, only the *PCF* subgroup exhibited a significant difference in *TCP* gene family size between the two species. Chromosomal localization and synteny analysis of the *G. paniculata TCP* gene family revealed that its gene expansion occurred after subfamily diversification (Figure 2e). The Class I subfamily (*PCF* clade) in *G. paniculata* underwent insufficient gene expansion post-diversification, resulting in fewer *TCP* gene family members compared to model species like *Arabidopsis thaliana* [33].

*TCP* transcription factors were demonstrated to play regulatory roles in the development of various plant organs, including leaf development [34,35,36,37], shoot branching [38,39], floral organ development [40,41,42] and root development [43,44]. In *Arabidopsis thaliana*, the TCP transcription factor family was extensively studied [5]. The *CIN* clade of *Arabidopsis*, *TCP2*, *TCP4*, *TCP10*, and *TCP24* were all targeted and suppressed by *miR319a* [45]. In Jaw-D mutants (*Arabidopsis miR319a* knockout), the expression levels of these five *TCP* genes were elevated, exhibiting multifaceted phenotypes including altered leaf shape, floral development defects, and delayed leaf senescence [45]. This module was also crucial for root development; when the *microRNA* target site on the *TCP4* sequence was mutated, root development was similarly suppressed [46]. In the *tcp4* single mutant, knocking down the other four *TCP* genes in the *CIN* clade enhances the impact on leaf shape phenotypes, indicating that genes in this *CIN* clade had diverse functions and exhibited partial redundancy [47,48].

This study confirmed that *GpTCP10* functioned as a negative regulator of flower size (Figure 5a,b), but its specific molecular mechanism of action remained to be further elucidated. Based on the present study and existing literature, we hypothesized that its regulatory mechanism may involve the following aspects. As a member of the *CIN* subfamily, *GpTCP10* may directly repress downstream genes that promote cell proliferation by binding to typical TCP cis-acting elements such as “GGNCCCAC” [49]. For example, in *Arabidopsis thaliana*, the *TCP* gene could inhibit the expression of the cell cycle gene *CYCLIN* [50]. We predicted that key genes involved in petal cell expansion or division, such as *EXPANSINS* and *CYCLIN B1*, may be transcriptionally suppressed by *GpTCP10* in transgenic lines, thereby resulting in reduced petal cell number or size. TCP proteins acted as critical hubs connecting developmental programs and hormonal signaling pathways. Previous studies had shown that the expression of multiple *TCP* genes was significantly regulated by plant hormones including abscisic acid (ABA) and methyl jasmonate (MeJA) [51,52]. We speculated that *GpTCP10* may restrict the growth potential of petals by interfering with the distribution or signal transduction of auxin or brassinosteroids in floral primordia. Further verification was required by detecting the contents of relevant hormones and the expression changes of their signaling-responsive genes in transgenic plants. In orchids, *CYC/TB1-like TCP* genes had been proven to regulate floral symmetry and organ identity by modulating floral organ identity genes such as B-class *MADS-box* genes [14,53]. Although the flowers of *G. paniculata* were radially symmetrical, it is a promising direction to explore whether *GpTCP10* indirectly affects the final morphology and size of petals through interactions with the core regulatory network of floral development. Emerging evidence suggests that *TCP* genes themselves may be finely modulated by post-transcriptional regulation, such as being targeted by microRNAs [45,54]. In the context of this study, the activity or expression level of *GpTCP10* may also be regulated by similar post-transcriptional mechanisms, which added another layer of complexity to the regulatory network of flower size.

Our research primarily focused on investigating the organ development-related functions of *GpTCP10*. Bioinformatics analysis indicated that *GpTCP10* belonged to the *CIN* clade (Figure 1a,b). In transgenic *Arabidopsis* over-expressing *GpTCP10*, we observed suppressed root length and lateral root number (Figure 3b–d), curled leaves (Figure 4a–d), and reduced floral organ size (Figure 5a–c), which was consistent with the functions of *CIN* subgroup *TCP* genes in *Arabidopsis* [55]. Furthermore, in *Phalaenopsis orchids*, *PeCIN8* exhibited a strong correlation with petal size: lower *PeCIN8* expression correlates with larger petals [53]. Our findings also demonstrated that *GpTCP10* expression was higher in smaller-flowered *G. paniculata* (Figure 5e). In *G. paniculata*, *GpTCP10* was specifically expressed in leaves and flowers (Figure 5e). Notably, specific expression of *GpTCP10* was observed in leaves and flowers of *G. paniculata*, whereas inhibited root growth (Figure 3b) and abnormal leaf curling were caused by its ubiquitous overexpression in transgenic *Arabidopsis* (Figure 4a,b). However, due to the lack of a stable genetic transformation system in *Gypsophila paniculata*, in-depth functional verification of *GpTCP10* in its native species is still to be performed in future studies.

## 4. Materials and Methods

### 4.1. Plant Materials

Two baby’s breath (*Gypsophila paniculata*) cultivars, D10 and D14, were used in this study. *Gypsophila paniculata* cultivar D10 exhibited flowers with an average diameter of about 10 mm. D10 exhibited smaller petals than cultivar D14, which has an average flower diameter of approximately 14 mm. These two cultivars were derived from the same parental line “Qianwanxing” and represented two distinct variants with similar genetic background but stable and distinguishable phenotypic traits. They were cultivated together in the same artificial greenhouse under natural daylight conditions at Yunnan Agricultural University, Kunming, China. The two *Gypsophila paniculata* cultivars were cultivated under uniform environmental conditions. Inter-row and intra-row spacing were both set to 15 cm to ensure consistent plant growth status. We collected root, stem, leaf, and flower samples, and immediately froze them in liquid nitrogen. These specimens were stored in −80 °C freezer until RNAs were extracted using the Trizol reagent (TransGen Biotech, Bejing, China) based on the manufacturer’s protocol. We evaluated the integrities of RNAs by examining the ratio of the optical density at 260 nm to 280 nm (OD 260/280) with an ultraviolet spectrophotometer (JIAPENG, Shanghai, China). RNA samples with 18S:28S ratio of 1.8–2.2 were regarded qualified and kept for further experiments.

The transgenic experiments used wild-type *Arabidopsis thaliana* (L.). Wild-type *Arabidopsis* seeds were obtained from the College of Landscape and Horticulture, Yunnan Agricultural University, Kunming, China. Both wild-type and transgenic *Arabidopsis* plants were grown in a peat/vermiculite mixture (1:1, *v*/*v*) under the same plant cultivation conditions described above.

### 4.2. Identification, Multiple Sequence Alignment, and Evolutionary Analysis of the GpTCP Gene Family

The genome, mRNA and protein sequences of *Gypsophila paniculata* were downloaded from the CNGB (China National GeneBank) Database by following the accession No. CNP0003304 reported in [31]. Genome, CDS, protein, and annotation information for *A. thaliana*, *Amborella trichopoda*, *Beta vulgaris*, *Chenopodium quinoa*, *Oryza sativa*, *Populus trichocarpa*, *Solanum lycopersicum* were obtained from the Arabidopsis Information Resource (TAIR) database [56] and National Center for Biotechnology Information (NCBI) website [57], respectively.

The eggNOG-mapper software (version 2.0) [58] were used to identify TCP family members in *G. paniculata* with the options of “-d virNOG –cpu 20 -m diamond”. After annotation using eggNOG-mapper, protein sequences belonging to the TCP family were filtered from the annotation results. The 17 *G. paniculata TCP* genes were named based on their similarity to the 24 *TCP* genes in *A. thaliana* (Supplementary Figure S1). The domains of the TCP proteins were analyzed using Pfam [59]. Protein physicochemical properties of the 17 TCPs identified were analyzed using the online website Expasy [60].

TCP family proteins from six other species besides *A. thaliana* were also annotated by eggmapper-NOG [58]. Multiple-sequence alignment of *G. paniculata* TCP proteins was performed using the ClustalW algorithm in MEGA software (v12) [61]. A phylogenetic tree was constructed for the 17 identified TCPs in *G. paniculata*, and TCP proteins from seven other species, including 24, 10, 15, 31, 24, 33, and 31 TCP proteins from *A. thaliana*, *A. trichopoda*, *B. vulgaris*, *C. quinoa*, *O. sativa*, *P. trichocarpa*, and *S. lycopersicum*, respectively, using the Neighbor-Joining and Maximum Likelihood method in MEGA, with 1000 bootstrap replicates. The resulting phylogenetic tree was visualized and enhanced and using the iTOL platform (v4) [62].

### 4.3. Gene Structure, Conserved Motif, and Promoter Element Analysis

The CDS and 2000 bp upstream promoter sequences of the *G. paniculata TCP* genes were extracted using Integrative Genomics Viewer (IGV, v2.18.2) [63]. Conserved protein motifs were analyzed using the MEME Suite online tool [64], with a focus on the identification of regulatory elements related to with organ development. Conserved domains were identified using the NCBI-CDD platform [65]. Gene structure diagrams, motif distribution maps, and protein domain diagrams were visualized using TBtools [66]. Promoter *cis*-regulatory element analysis was performed using the PlantCARE platform [67]. These elements were visualized using the TBtools CSimple BioSequence Viewer [66].

### 4.4. Chromosome Localization and Synteny Analysis

Using the genome annotation GFF file, the chromosomal localization information for the *TCP* gene in *G. paniculata* was extracted [31]. The chromosomal localization information for the *TCP* genes in *Arabidopsis thaliana* were extracted using the same method [56]. The TBtools software (v2.446) was then employed to visualize their positional distributions [66]. The MCScanX one-step toolkit integrated within TBtools was used to identify homology and synteny relationships between *G. paniculata* and *Arabidopsis thaliana* [66]. The cross-species synteny map was generated using TBtools’ dual homology mapping functionality [66]. Using only the chromosomal localization data of *G. paniculata TCP* genes, TBtools performed intraspecific colinearity analysis for *G. paniculata* [66]. The non-synonymous substitution rate (*Ka*) and synonymous substitution rate (*Ks*) for homologous gene pairs were calculated and visualized. Divergence time (*T*) was estimated using the formula T=0.5Ks/λ, where λ = $3.02\times 10^{-9}$ substitutions per site per year [68].

### 4.5. Over-Expression Vector Construction

The procedure for constructing the *GpaTCP10* over-expression vector was as follows. The pCAMBIA1391 vector (NovoPro, Shanghai, China) was double-digested with restriction enzymes EcoRI and SalI, and linearised vector fragments were recovered from the gel. Using cDNA as a template, the full-length CDS sequence of *GpaTCP10* was amplified via PCR with specific primers containing restriction sites (sequences provided in Supplementary Table S1). Following gel electrophoresis verification of the correct band size, the target fragment was recovered. The linearised vector and *GpaTCP10* CDS fragment was ligated using homologous recombination technology. The ligation product was transformed into DH5α chemotrophic competent cells (Weidi Bio, Shanghai, China), and successful vector-target fragment ligation was verified via colony PCR. Plasmid extraction was performed from DH5α strains harbouring positive clones. The resulting plasmid was submitted for Sanger sequencing (Sangon Biotech, Shanghai, China) to verify the absence of mutations in the inserted fragment. The final recombinant plasmid was obtained and stored at −20 °C.

### 4.6. Arabidopsis Transformation and Phenotype Identification

This study employed the inflorescence-dipping method, where the over-expression vector was Agrobacterium-mediated into *A. thaliana*. The successfully constructed over-expression vector was transformed into Agrobacterium GV3101 (Weidi Bio, Shanghai, China) for cultivation. The Agrobacterium cells harboring the plasmid were cultured and collected. The cells were then re-suspended in a permeabilization buffer to an $OD_{600}$ of 0.4 to 0.6. This buffer was composed of sterile water containing 5% sucrose, sterilized at 121 °C for 20 min, and supplemented with 0.02% to 0.03% SiLwet L-77. Immerse *Arabidopsis* inflorescences in Agrobacterium infection solution for 30 s, then transfer them to normal light conditions after 24 h of dark cultivation. Both wild-type and *GpTCP10* over-expressing lines were sown in 1/2 MS medium. Harvest transgenic *Arabidopsis* seeds approximately 30 days later and screen using Hygromycin B (Coolaber, Beijing, China). When the four selected T1-generation *Arabidopsis* plants developed 7–8 true leaves, basal leaves were collected for PCR-based positive identification. Seeds harvested from the four T1-generation plants were sown, and T2-generation *Arabidopsis* plants underwent phenotypic recording and RT-qPCR validation.

The T3-generation and WT plants were cultivated in a light-controlled incubator for phenotype observation. We analyzed 36 wild-type plants, 29 OE2 line plants, 24 OE4 line plants, and 36 OE7 line plants in the phenotypic comparison experiment. When measuring the length of the primary roots, two OE4 plants and one OE7 plant had indistinguishable primary roots and were therefore excluded from the measurement. For flower size measurements, we selected 16 wild-type plants, 11 OE2 plants, 12 OE4 plants, and 10 OE7 plants. All flowers were sampled on the same day to guarantee uniform flowering openness, consistent growth conditions, and synchronized flowering periods. Therefore, the real numbers of samples used for data collection are smaller than the total cultivated plants. The specific data from phenotypic observations could be found in Supplementary Tables S4–S8.

Seedings of four *Arabidopsis thaliana* lines were surface sterilized and sown simultaneously on 1/2 MS medium. Following 72 h of vernalization, the seedlings were transferred to light incubator in the next 30 days with 16 h light/8 h dark cycle, at the temperate of 22±1°C. For phenotypic analysis, primary root length and lateral root number were recorded in 13-day-old seedlings of the *Arabidopsis* lines. Subsequently, 14-day-old seedlings were transplanted into a vermiculite mixture to allow for continued growth. The leaf morphology of the uppermost whorl at 21-day-old seedlings; and the flower morphology of fully opened flowers at 30-day-old seedlings.

When measuring flower sizes for D10 and D14, we conducted 10 biological replicates and 3 technical replicates for each flower’s diameter measurement. All baby’s breath flowers were harvested on the same day, i.e., the 21st day after flower bud formation. The individual values presented in the statistical graph represented the average of the 3 technical replicates. The specific data from phenotypic observations could be found in Supplementary Table S9.

Morphological variations in leaf surfaces were assessed by measuring the orthographic projection area of the adaxial epidermis. Additionally, the length of the primary vein on the abaxial surface was measured from the leaf base margin to the distal tip. IMAGEJ (v1.54g) software [69] was utilized for all area and length quantifications.

### 4.7. Validating the Differentially Expressed Genes with qRT-PCR

Total RNAs were isolated from root, stem, leaf, and flower samples of two varieties of baby’s breath (D10 and D14). The target gene *GpTCP10* was analyzed, and *GpActin* was used as the internal reference gene for normalization [31]. Additionally, in the qRT-PCR validation experiments conducted on *Arabidopsis* leaf samples, the target gene *GpTCP10* was analyzed, and *AthActin* was used as the internal reference gene for normalization [57]. RNA extraction was performed using a plant RNA extraction kit (TransGen Biotech, Beijing, China), following the manufacturer’s instructions. First-strand cDNA was synthesized from 1 μg of total RNA using the FastKing gDNA Dispelling RT SuperMix reagent kit (TIANGEN BIOTECH, Bejing, China) in a 20-μL reaction volume. qRT-PCR was performed using Taq SYBR® Green qPCR Mix (LABLEAD, Beijing, China) with the procedure of 30 s at 95 °C followed by 40 cycles of 10 s at 95 °C, 10 s at 60 °C and 30 s at 72 °C on CFX Connect Real-Time PCR System (Bio-Rad, Hercules, CA, USA). *GpaTCP* was derived from annotations of the reference genome that had already been reported [31]. Each sample was analyzed with three biological replicates and three technical replicates. Relative gene expression levels were calculated using the ($2^{-\Delta \Delta Ct}$) method [70]. Statistical significance was determined by *t*-test. Primer sequences used in RT-qPCR are listed in Supplementary Table S2.

### 4.8. Statistical Analysis

The values of different groups were compared with *t*-tests. The numbers of lateral roots at the day 13 from seedings of *GpTCP10* over-expressing transgenic and WT *Arabidopsis* lines were compared with the Wilcoxon rank-sum test. *p*-values of <0.05 and <0.01 were considered statistically significant and extremely significant, respectively. Graphical representations were generated using GraphPad Prism (v8.0.1) (GraphPad Software, San Diego, CA, USA).

## 5. Conclusions

We identified 17 members of the *TCP* gene family in *G. paniculata* through genomic analysis and phylogenetic analysis (Table 1 and Figure 1). Chromosomal colinearity analysis of *G. paniculata TCP* family members revealed that gene expansion occurred after subfamily differentiation (Figure 2e). The *Arabidopsis GpTCP10* over-expressing line exhibited phenotypes including shorter roots, reduced lateral root number (Figure 3b–d), leaf curling (Figure 4a–d), yellow flower and smaller flower size (Figure 5a,b). Our results indicated that *GpTCP10* was specifically expressed in *G. paniculata* leaves and flowers, with significantly higher *GpTCP10* expression in the smaller-flowered variety (Figure 5e), consistent with the phenotype observed in *Arabidopsis GpTCP10* over-expressing lines (Figure 5a–c). In summary, our results suggested that *GpTCP10* functioned as a negative regulator of organ growth and development in *G. paniculata*.

## Figures and Tables

**Table 1:** The physicochemical properties of GpTCP family protein in *G. paniculata*.

Name	Gene ID	Homolog	AA	MW	pI	II	GRAVY	AI	SP	TH	SL
GpTCP20b	Gpan01g00759	AtTCP20	231	24,918.63	7.23	57.40	−0.686	70.22	NO	0	Nucleus
GpTCP20a	Gpan16g00434	AtTCP20	234	25,430.94	7.10	42.05	−0.916	56.37	NO	0	Nucleus
GpTCP8	Gpan08g01775	AtTCP8	185	19,205.24	7.06	53.25	−0.700	58.59	NO	0	Nucleus
GpTCP15	Gpan08g01206	AtTCP15	466	48,924.26	7.01	61.19	−0.722	51.55	NO	0	Nucleus
GpTCP14	Gpan09g00267	AtTCP14	431	46,228.92	6.76	53.78	−0.874	49.44	NO	0	Nucleus
GpTCP11	Gpan16g00380	AtTCP11	227	24,530.58	8.72	69.92	−0.401	71.32	NO	0	Nucleus
GpTCP3a	Gpan03g01596	AtTCP3a	461	49,013.24	8.64	59.09	−0.594	63.97	NO	0	Nucleus
GpTCP3b	Gpan15g00412	AtTCP3b	420	45,402.64	6.70	56.38	−0.755	58.29	NO	0	Nucleus
GpTCP10	Gpan06g00621	AtTCP10	374	40,080.62	6.39	37.30	−0.755	59.47	NO	0	Nucleus
GpTCP4	Gpan01g00196	AtTCP4	202	22,751.24	4.90	48.14	−0.715	68.07	NO	0	Nucleus
GpTCP5a	Gpan08g01151	AtTCP5	365	40,816.98	7.02	58.33	−0.777	65.97	NO	0	Nucleus
GpTCP5b	Gpan11g00693	AtTCP5	337	37,829.11	7.89	55.21	−0.754	71.19	NO	0	Nucleus
GpTCP5c	Gpan11g00727	AtTCP5	384	42,493.80	7.37	56.86	−0.782	65.52	NO	0	Nucleus
GpTCP1	Gpan01g00724	AtTCP1	329	37,398.00	8.41	50.67	−0.831	67.54	NO	0	Nucleus
GpTCP18	Gpan17g00108	AtTCP18	392	44,936.65	9.40	57.42	−0.973	69.16	NO	0	Nucleus
GpTCP12a	Gpan03g00539	AtTCP12	263	29,767.23	9.34	37.90	−1.164	53.42	NO	0	Nucleus
GpTCP12b	Gpan12g00867	AtTCP12	311	35,357.11	6.77	50.93	−1.208	50.80	NO	0	Nucleus

AA, number of amino acids; MW(Da), molecular weight; pI, theoretical isoelectric point; II, instability index; GRAVY, grand average of hydropathicity; AI, aliphatic index; SP, signal peptide; TH, transmembrane helix; SL, subcellular location prediction.

## Data Availability

The original contributions presented in this study are included in the article/Supplementary Materials. Further inquiries can be directed to the corresponding authors.

## Supplementary Materials

The following supporting information can be downloaded at https://www.mdpi.com/article/10.3390/plants15060949/s1, Figure S1: Phylogenetic analysis of TCP proteins from *Arabidopsis thaliana* and *Gypsophila paniculata*; Figure S2: Phylogenetic analysis of TCP proteins from *Gypsophila paniculata*, *Arabidopsis thaliana*, *Amborella trichopoda*, *Beta vulgaris*, *Chenopodium quinoa*, *Oryza sativa*, *Populus trichocarpa*, *Solanum lycopersicum*; Table S1: Primer used for cloning GpTCP10 in *Gypsophila paniculata*; Table S2: The primers used to validate selected genes *GpTCP10* that are differently expressed in *A. thaliana* and *Gypsophila paniculata*; Table S3: Expression of *GpTCP10* gene in diffrernt Arabidopsis line ($2^{-\Delta \Delta Ct}$); Table S4: Comparisons of root lengths at the day 13 from seedings of *GpTCP10* over-expressing transgenic and WT Arabidopsis lines; Table S5: Comparisons of the numbers of lateral roots at the day 13 from seedings of *GpTCP10* over-expressing transgenic and WT Arabidopsis lines; Table S6: Comparisons of projected areas of the adaxial leaves at the day 30 from seedings in *GpTCP10* over-expressing transgenic and WT Arabidopsis lines; Table S7: Comparisons of the lengths of the primary leaf veins on abaxial leaves at the day 30 from seedings of *GpTCP10* over-expressing transgenic and WT Arabidopsis lines; Table S8: Comparisons of flower lengths at the day 30 from seedings of *GpTCP10* over-expressing transgenic and WT Arabidopsis lines; Table S9: Comparisons of single flower diameters at day 21 after flower bud formation for two cultivars of *Gypsophila paniculata*, D10 and D14; Table S10: Expression of *GpTCP10* gene in diffrernt tissues of two varieties of *Gypsophila paniculata* ($2^{-\Delta \Delta Ct}$); Table S11: Expression of *GpTCP10* gene in diffrernt tissues of D10 ($2^{-\Delta \Delta Ct}$); and Table S12: Expression of *GpTCP10* gene in diffrernt tissues of D14 ($2^{-\Delta \Delta Ct}$).

## Abbreviations

The following abbreviations are used in this manuscript:

WT	Wild-type *Arabidopsis thaliana*
OE	*GpTCP10* over-expressing *Arabidopsis thaliana* line
AA	Number of amino acids
MW	Molecular weight
pI	Theoretical isoelectric point
II	Instability index
GRAVY	Grand average of hydropathicity
AI	Aliphatic index
SP	Signal peptide
TH	Transmembrane helix
SL	Subcellular location prediction
