# Supplementary material for: Genome-Wide Identification of the TCP Gene Family and Functional Analysis of Gypsophila paniculata GpTCP10 in Regulating Organ Development of Transgenic Arabidopsis
Source: Plants (Basel). 2026 Mar 19;15(6):949. doi: 10.3390/plants15060949 (PMC13030823; doi:10.3390/plants15060949)
Supplement: Supplementary file 1 [file plants-15-00949-s001.zip › supplementary figures.pdf]

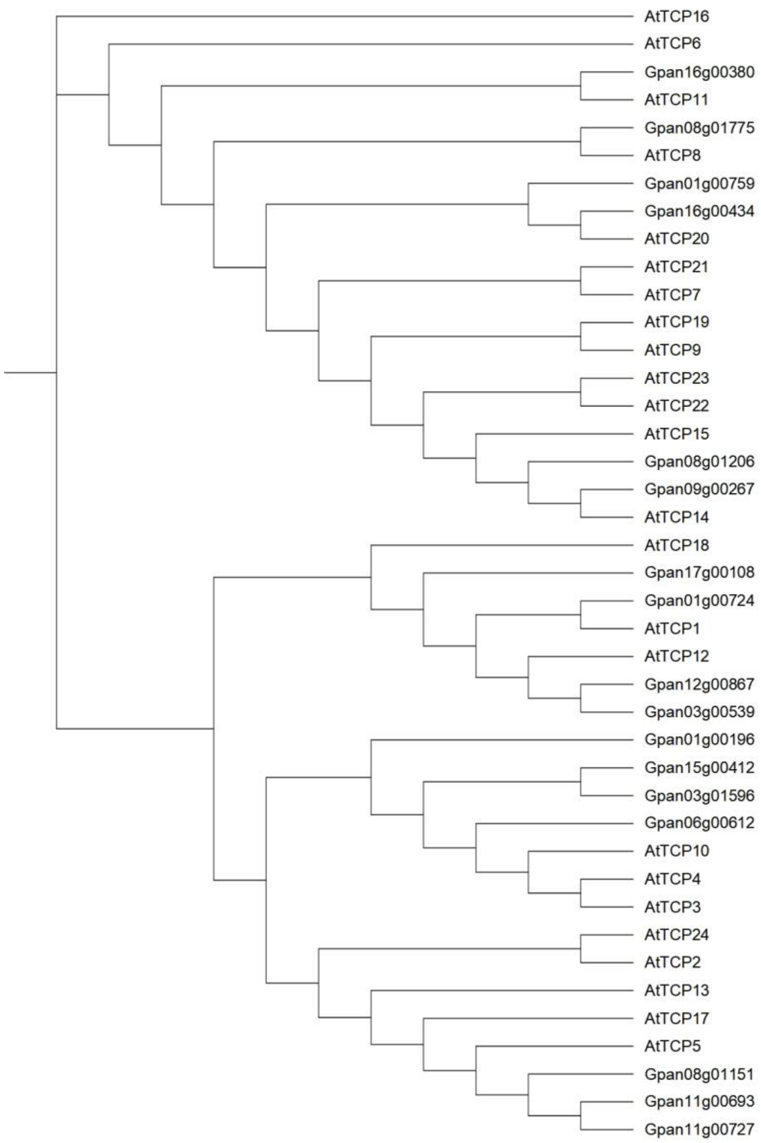

Figure S1. Phylogenetic analysis of TCP proteins from *Arabidopsis thaliana* and *Gypsophila paniculata*. The phylogenetic tree was constructed using the Neighbor-Joining method, with 1000 bootstrap replicates, using MEGA (v12).

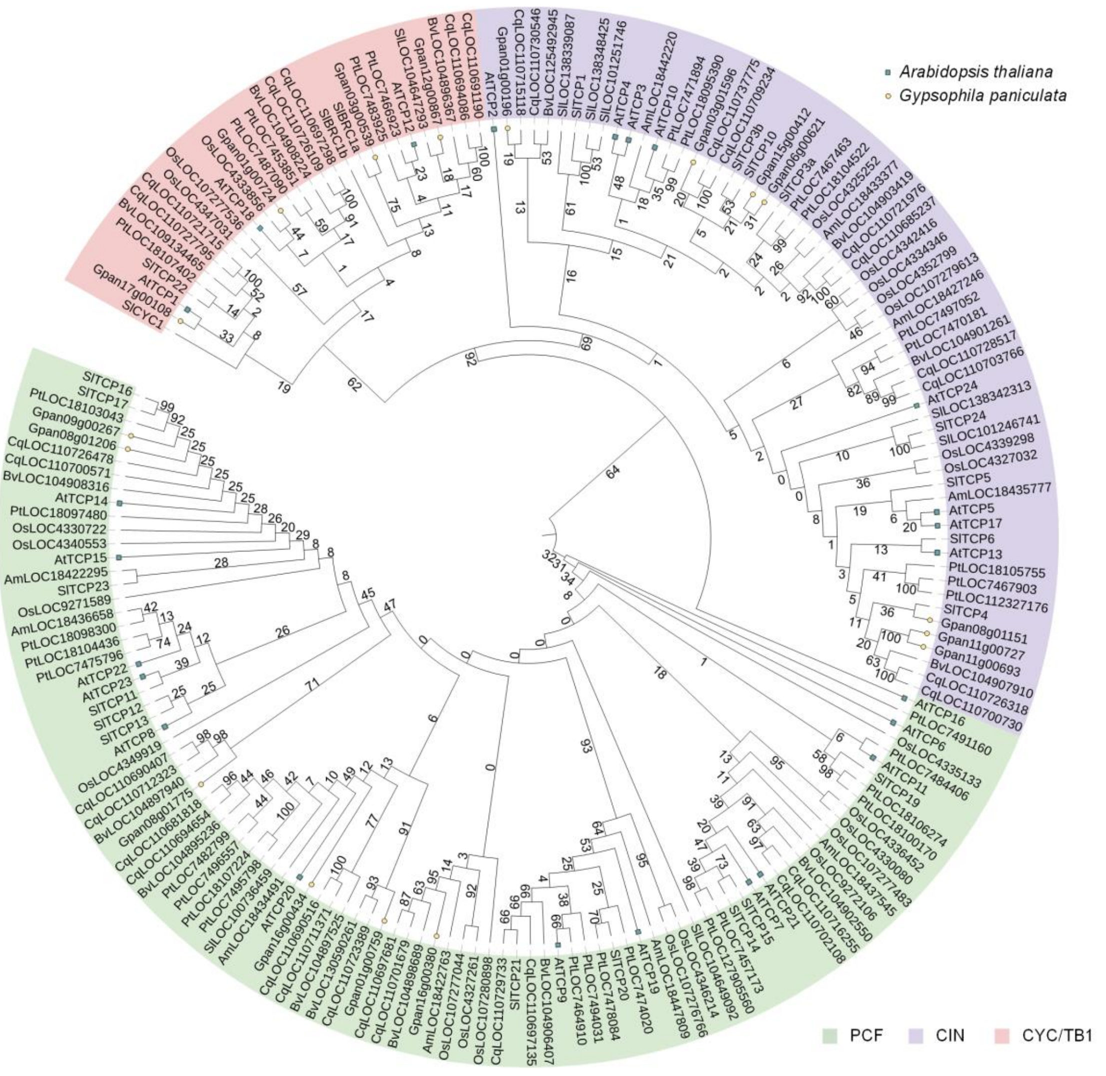

Figure S2. Phylogenetic analysis of TCP proteins from *Gypsophila paniculata*, *Arabidopsis thaliana*, *Amborella trichopoda*, *Beta vulgaris*, *Chenopodium quinoa*, *Oryza sativa*, *Populus trichocarpa*, *Solanum lycopersicum*. The phylogenetic tree was constructed using the Neighbor-Joining method, with 1000 bootstrap replicates, using MEGA (v12).
